# Permanent stoma: a quality outcome in treatment of rectal cancer and its impact on length of stay

**DOI:** 10.1186/s12893-021-01166-7

**Published:** 2021-03-25

**Authors:** Riccardo Lemini, Iktej S. Jabbal, Krystof Stanek, Shalmali R. Borkar, Aaron C. Spaulding, Scott R. Kelley, Dorin T. Colibaseanu

**Affiliations:** 1 Division of Colon and Rectal Surgery, Mayo Clinic, 4500 San Pablo Rd, Jacksonville, FL 32224 USA; 2 Department of Health Sciences Research, Mayo Clinic, Jacksonville, FL USA; 3 Division of Colon and Rectal Surgery, Mayo Clinic, Rochester, MN USA

**Keywords:** Rectal cancer, Stoma, Outcomes, Surgery, Length of stay, Colorectal surgery, Disparities

## Abstract

**Background:**

This study aimed to identify socioeconomic predictors of permanent stoma in rectal cancer treatment and examine its association with length of stay at the treatment facility.

**Methods:**

Rectal cancer patients who underwent elective surgery between January 2015 and December 2018 were identified from the Agency for Health Care Administration Florida Hospital Inpatient Discharge Dataset. Multivariate regression models were utilized to identify demographic and socioeconomic factors associated with receiving a permanent stoma as well as the associated length of stay of these patients.

**Results:**

Of 2630 rectal cancer patients who underwent surgery for rectal cancer, 21% had a permanent stoma. The odds of receiving permanent stoma increased with higher Elixhauser score, metastatic disease, advanced age, having open surgery, residence in Southwest Florida, and having Medicaid insurance or no insurance/self-payers (p < 0.05). Patients with a permanent stoma had a significantly extended stay after surgery (p < 0.001).

**Conclusions:**

Patients with a permanent stoma following cancer resection were more likely to have open surgery, had more comorbidities, and had a longer length of stay. Having permanent stoma was higher in patients living in South West Florida, patients with Medicaid insurance, and in the uninsured. Additionally, the payer type significantly affected the length of stay.

**Supplementary Information:**

The online version contains supplementary material available at 10.1186/s12893-021-01166-7.

## Background

Colorectal cancer is the third most common type of cancer worldwide and also the third most common cause of mortality related to cancer in the United States [1]. Surgical resection is the mainstay of treatment in patients with rectal cancer. Deliberate efforts in improving surgical techniques, the advent of robotic surgery, and additional minimally invasive training of surgeons have all worked towards the patients’ benefit. Though stoma was never something that patients sought, these additional -purposeful- efforts have allowed patients to have more options. However, in some cases, a stoma (temporary or permanent) is necessary as part of the rectal cancer treatment [2]. The decision to proceed with either type of stoma is complex and multifactorial. The patient's general status, including comorbidities, inflammation, fecal incontinence, and other clinical factors, influence the decision to create an ostomy. Ideally, both clinical and technical factors should decide the type and approach of surgery [3]. These factors, in addition to the stage of the cancer, locoregional invasion, and expertise of the surgeon, should be the only determinants whether a patient is receiving a stoma or not. However, several authors have found disparities in the receipt of care associated with sex, age, race, health insurance, and other sociodemographic and geographic factors [4–7]. Presently, no current literature effectively studies the impact of all the elements together on receipt of stoma in rectal cancer patients. Given the importance of all of these aspects in delivering optimal care, it is crucial to determine how each might influence the care progress and the receipt of a permanent stoma.

Thus, the purpose of this study was to evaluate the factors associated with the odds of having a permanent stoma following resection in patients with rectal cancer. Besides, we assessed the factors associated with a longer length of stay (LOS) at the treatment facility in the same cohort of individuals.

## Methods

**Fig. 1:**
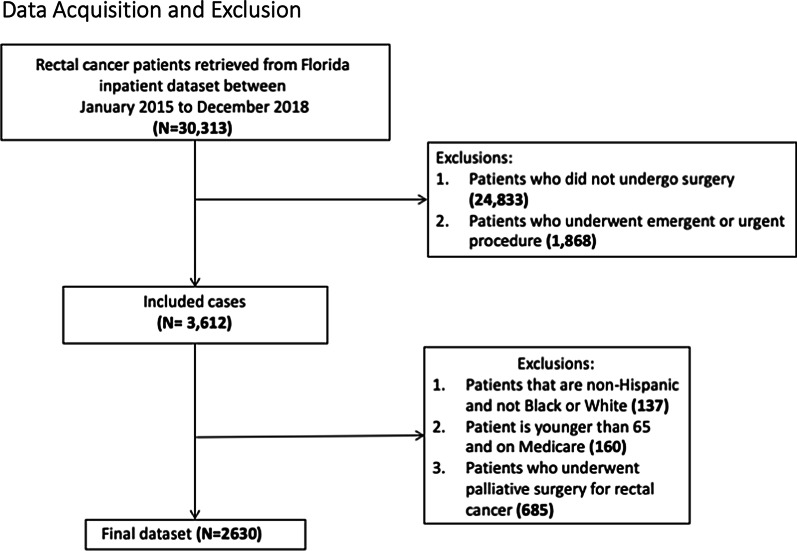
Flow diagram for study selection

Patients with rectal cancer undergoing surgery between January 2015 and December 2018 were identified from the Florida Agency for Health Care Administration (AHCA)—2015–2018 Hospital Inpatient dataset using the ICD-10 codes C19 and C20 [8]. The Florida inpatient dataset is an administrative dataset that includes all patient admissions from hospitals in Florida. It includes non-patient identifiable data for a patient’s admission and provides diagnosis and procedure codes and patient demographic information. Using ICD 10 codes, we identified patients with a diagnosis of rectal cancer (n = 30,313) and only included patients who underwent “resection of tumor,” “resection with colostomy,” or “resection with ileostomy” for the treatment of rectal cancer (n = 5480) (see Additional file 1: Table S1). Patients who underwent procedures identified as emergent or urgent (n = 1,868), those who were non-Hispanic non-white, or non-Hispanic not black (n = 137), those who were under 65 and on Medicare insurance (n = 160) as well as patient who underwent palliative surgery for rectal cancer (n = 685) were excluded. This resulted in a dataset with 2630 observations (see Fig. 1). Due to the de-identified and publicly available nature of the dataset, this research was categorized as exempt by the Institutional Review Board.

### Dependent variable

The primary dependent variable for this study was the receipt of a permanent stoma. Patients who underwent colostomy with resection were grouped as having “permanent stoma.” Patients who underwent ‘only resection of rectal cancer’ or ‘resection with ileostomy’ were included in the group “no permanent stoma” as either a stoma was not created in these surgeries or a temporary stoma was created to allow the anastomosis to heal completely.

Also, LOS at the surgical facility was studied as the second dependent variable. LOS was available as a continuous variable with distribution skewed towards the right. Logarithmic transformation of this variable was utilized to normalize the distribution, and the range was limited to exclude patients who stayed more than 14 days in the surgical facility.

### Independent variables

Patient characteristics included in the analysis were demographics such as age, sex, and race/ethnicity. Race and ethnicity were categorized as White, African American, and Hispanic or Latino. Patient’s payer type, rural/urban location, and residential region in Florida were added to the model to adjust for the influence of the patient's social factors on the LOS at the surgical facility. Payer type included Commercial, Medicaid, Medicare, Medicare Managed Care, and Other, which includes both self-payers and uninsured. We also adjusted for the comorbidity score using the Elixhauser Scoring system [9]. We created three categories indicating the presence of 0, 1–2, greater than 3 comorbidities. Metastatic status of cancer and obesity may significantly influence the patient's likelihood of receiving a permanent stoma and LOS at a treatment facility and were included separately in the analysis. The type of surgical approach, open/minimally invasive, was also adjusted for in the study. The two approaches are quite different and may bias the influence on the LOS after surgery.

### Statistical analysis

We first conducted a bivariate analysis to examine the factors associated with having a permanent stoma created to treat rectal cancer. Pearson chi-square test and Kruskal–Wallis test were used to compare categorical and continuous variables, respectively. Multivariate logistic regression analysis was conducted to understand patients' characteristics more likely to be associated with getting a permanent stoma. The logarithmic transformation of the LOS variable met the assumptions of linear regression. Multivariate linear regression was then performed to compare the LOS at the surgical facility for the rectal cancer patients after adjusting for permanent stoma and other covariates.

## Results

This study included 2,630 patients who underwent surgical procedures to treat rectal cancer between 2015 and 2018 in 182 Florida hospitals; 552 (21%) out of these had a permanent stoma. The overall patient characteristics are summarized in Table 1. The majority of the population was male (1576, 59.9%). While the mean age of the study population was 62.8 years with a standard deviation of 12.2 years, the mean age of patients who ultimately had a permanent stoma was significantly higher than those who did not (p < 0.01). The majority of the population was White (1946, 75.9%), followed by Hispanic/Latino (443, 17.3%) and African-American (176, 6.9%). Additionally, there is a significant association (p = 0.02) between race and permanent ostomy creation. There were 1,872 (71.2%) patients with 1 or more comorbidities, 547 (20.8%) patients with metastatic cancer, and 360 (13.7%) were found to be obese. With an increase in the Elixhauser score, the percentage of patients undergoing surgery for permanent stoma proportionately increased (p = 0.01). Similarly, metastasis was associated with a higher chance of having a permanent stoma (p < 0.01). Though most of the rectal cancer patients in the study population had commercial insurance (47.4%) compared to other insurances, the commercially insured patients had a lower rate of receiving a permanent stoma, while those with no insurance or self-pay had a higher rate of receiving a permanent stoma. Additionally, there are significant associations between insurance type and receipt of permanent stoma (p < 0.01). The geographic location of residence in Florida was significantly associated with receiving a permanent stoma (p = 0.05). Year of the surgery was not associated with receiving a permanent stoma (p = 0.36), and a larger proportion of surgeries occurred in 2016 (n = 951, 36.2%) compared to other years. The mean and median LOS was significantly longer in patients with permanent stoma surgery (mean 6.6 days, std. dev. 2.9 vs. mean 5.4 days, std. dev. 2.8). Finally, in our cohort of patients undergoing management for rectal cancer, more patients underwent open surgery instead of Minimally invasive surgery, with a greater proportion of patients undergoing open surgery in the permanent ostomy group (p < 0.01).

**Table 1:** Characteristics of patients who had permanent stoma surgery for the treatment of rectal cancer

Variable	Permanent Stoma		Total (N = 2630)	p-value
	Yes (N = 552)	No (N = 2078)		
Sex				
Females	206 (37.3%)	848 (40.8%)	1054 (40.1%)	0.14^1^
Males	346 (62.7%)	1230 (59.1%)	1576 (59.9%)	
Age				
Median (Range)	66.0 (32.0, 94.0)	63.0 (22.0, 97.0)	63.0 (22.0, 97.0)	< 0.01^2^
Mean (SD)	65.1 (12.0)	62.2 (12.2)	62.8 (12.2)	
Race				
White	427 (77.3%)	1519 (73.09%)	1946 (74.0%)	0.02^1^
African American	44 (7.9%)	132 (6.4%)	176 (6.7%)	
Hispanic or Latino	73 (13.2%)	370 (17.8%)	443 (16.8%)	
Missing	8	57	65	
Elixhauser Score				
0	134 (24.2%)	624 (30.0%)	758 (28.8%)	0.01^1^
1–2	257 (46.5%)	984 (47.3%)	1241 (47.2%)	
3–5	161 (29.1%)	470 (22.6%)	631 (24.0%)	
Metastatic cancer				
No	401 (72.6%)	1682 (80.9%)	2083 (79.2%)	< 0.01^1^
Yes	151 (27.3%)	396 (19.0%)	547 (20.8%)	
Obesity				
No	479 (86.8%)	1791 (86.1%)	2270 (86.3%)	0.72^1^
Yes	73 (13.2%)	287 (13.8%)	360 (13.7%)	
Patient Payer				
Medicare	147 (26.6%)	416 (20.0%)	563 (21.4%)	< 0.01^1^
Medicare Managed Care	121 (21.9%)	407 (19.5%)	528 (20.1%)	
Medicaid	46 (8.3%)	123 (5.9%)	169 (6.4%)	
Commercial	205 (37.1%)	1041 (50.0%)	1246 (47.4%)	
Self-Pay/Uninsured	33 (6.0%)	91 (4.3%)	124 (4.7%)	
Patient Region				
Southwest Florida	108 (19.6%)	313 (15.0%)	421 (16.7%)	0.05^1^
Northeast Florida	48 (8.7%)	233 (11.2%)	281 (11.1%)	
Northwest Florida	36 (6.5%)	108 (5.1%)	144 (5.7%)	
Southeast Florida	101 (18.3%)	371 (17.9%)	472 (18.7%)	
Central Florida	97 (17.6%)	384 (18.4%)	481 (19.0%)	
South Florida	41 (7.4%)	254 (12.2%)	295 (11.7%)	
West Central Florida	88 (15.9%)	343 (16.5%)	431 (17.1%)	
Missing	33	72	105	
Patient county				
Rural	37 (6.7%)	104 (5.0%)	141 (5.6%)	0.09^1^
Urban	482 (87.3%)	1902 (91.5%)	2384 (94.4%)	
Missing	33	72	105	
Year				
2015	43 (7.8%)	184 (8.9%)	227 (8.6%)	0.36^1^
2016	192 (34.8%)	759 (36.5%)	951 (36.2%)	
2017	148 (26.8%)	576 (27.7%)	724 (27.5%)	
2018	169 (30.6%)	559 (27.0%)	728 (27.7%)	
Length of Stay (Days)				
Median (Range)	6.0 (1.0, 14.0)	5.0 (1.0, 14.0)	5.0 (1.0, 14.0)	< 0.01^2^
Mean (SD)	6.6 (2.9)	5.4 (2.8)	5.6 (2.8)	
Surgical approach				
Open surgery	306 (55.4%)	868 (41.2%)	1174 (47.6%)	< 0.01^1^
Minimally invasive surgery	196 (35.5%)	1097 (52.8%)	1293 (52.4%)	
Missing	50	113	163	

^1^Chi-Square p-value

^2^Kruskal–Wallis p-value

Logistic regression models for receipt of a permanent vs. non-permanent stoma (Table 2) showed that the odds of having a permanent stoma surgery increased with age (OR 1.02, 95% CI 1.00–1.03). The odds of receiving a permanent stoma were significantly higher in patients who had more than 3 comorbidities (OR 1.37, 95% CI 1.01–1.87) and those who had metastatic disease (OR 1.74, 95% CI 1.36–2.22). Compared to patients with commercial insurance, those who had Medicaid insurance (OR 1.80, 95% CI 1.18–2.74) or had no insurance/self-payers (OR 1.83, 95%CI 1.12–3.02) were more likely to have a permanent stoma formed at surgery. Additionally, compared to those residing in Northeast Florida, patients residing in Southwest Florida (OR 1.62, 95% CI 1.06–2.46) were more likely to get a permanent stoma. Finally, patients receiving an open surgery compared to MIS were more likely to receive a permanent stoma (OR 1.88, 95% CI 1.52–2.33).

**Table 2:** Multivariate analysis of factors associated with odds of patients having a permanent stoma surgery

Odds ratio estimates				
Effect	Point estimate	95% Wald confidence limits		p-value
Male	1.20	0.96	1.48	0.10
Age	1.02	1.01	1.03	0.01
Race				
White	–	–	–	–
African American	1.03	0.67	1.58	0.89
Hispanic or Latino	0.97	0.68	1.38	0.85
Elixhauser score				
Elixhauser score 0	–	–	–	–
Elixhauser score 1–2	1.10	0.84	1.43	0.50
Elixhauser score 3–5	1.37	1.01	1.87	0.05
Metastatic cancer	1.74	1.37	2.23	< 0.01
Obese	0.90	0.66	1.23	0.50
Patient Payer				
Commercial	–	–	–	–
Medicaid	1.80	1.18	2.74	0.01
Medicare	1.18	0.82	1.71	0.38
Medicare Managed Care	0.97	0.67	1.42	0.88
Self-Pay/Uninsured	1.84	1.12	3.02	0.02
Patient Region				
Northeast Florida	–	–	–	–
Central Florida	1.28	0.84	1.95	0.26
Northwest Florida	1.44	0.84	2.47	0.19
South Florida	0.83	0.47	1.46	0.52
Southeast Florida	1.34	0.87	2.06	0.18
Southwest Florida	1.62	1.06	2.46	0.03
West Central Florida	1.32	0.86	2.03	0.21
Year				
2015	–	–	–	–
2016	1.13	0.76	1.69	0.55
2017	1.18	0.78	1.78	0.43
2018	1.21	0.80	1.82	0.37
Urban	0.68	0.43	1.07	0.09
Open Surgery	1.88	1.52	2.33	< 0.01

Additional factors associated with a longer length of stay in the hospital (Table 3) were male gender (p = 0.01), presenting with comorbidities (p = 0.01 and p < 0.01), and metastatic cancer (p = 0.02), and receiving open surgery (p < 0.01). African American patients (p = 0.01), those covered by Medicaid (p < 0.01), and patients residing in Central (p = 0.01), Southwest (p = 0.01), Southeast (p = 0.02), and West Central Florida (p = 0.03) also spent a significantly longer time at the hospital. However, patients receiving surgeries in more recent years experienced a shorter length of stay compared to patients receiving surgery in 2015 (p = 0.01). In addition, patients receiving a permanent stoma were also associated with a longer length of stay.

**Table 3:** Multivariate analysis predicting the length of stay in patients receiving rectal cancer surgery

Parameter	Estimate	Standard Error	t value	p-value
Permanent Stoma	1.02	0.14	7.32	< 0.01
Male	0.33	0.11	2.98	0.01
Age	− 0.02	0.01	− 0.29	0.77
Race				
White	–	-	-	-
African American	0.68	0.23	3.02	0.01
Hispanic or Latino	− 0.06	0.18	− 0.33	0.74
Elixhauser score				
Elixhauser score 0	–	–	–	–
Elixhauser score 1–2	0.42	0.14	3.14	0.01
Elixhauser score 3–5	1.39	0.17	8.42	< 0.01
Metastatic cancer	0.33	0.134	2.36	0.02
Obese	0.31	0.16	1.91	0.06
Patient Payer				
Commercial	–	–	–	–
Medicaid	0.99	0.23	4.30	< 0.01
Medicare	0.09	0.20	0.44	0.66
Medicare Managed Care	0.21	0.20	1.08	0.28
Self-Pay/Uninsured	0.47	0.28	1.67	0.10
Patient Region				
Northeast Florida	–	–	–	–
Central Florida	0.56	0.22	2.59	0.01
Northwest Florida	− 0.27	0.29	− 0.95	0.34
South Florida	− 0.08	0.27	− 0.28	0.78
Southeast Florida	0.71	0.22	3.20	0.01
Southwest Florida	0.49	0.22	2.27	0.02
West Central Florida	0.47	0.22	2.12	0.03
Urban	− 0.07	0.25	− 0.28	0.78
Year				
2015	–	–	–	–
2016	− 0.40	0.21	− 1.96	0.05
2017	− 0.57	0.21	− 2.69	0.01
2018	− 0.76	0.21	− 3.57	0.01
Open surgery	1.23	0.11	10.95	< 0.01

## Discussion

Our analysis highlights several clinical and socioeconomic factors associated with the odds of receiving a permanent stoma when undergoing surgery for rectal cancer. These include age, being uninsured/self-pay or having Medicaid insurance, a higher number of comorbidities, metastatic disease, open surgery, and having surgery in South West Florida compared to the other parts of Florida.

Surgical therapy for rectal cancer has, over the past 100 years, evolved from more radical operations to modern sphincter-preserving techniques. These changes have primarily been driven by an increased understanding of the pathophysiology of rectal cancer, multimodal treatment, improved technology, surgical innovation, and by surgeons placing greater importance on the patients’ quality of life [2]. The oncologic and functional outcomes of preserving the intestinal continuity, however, continue to be a matter of ongoing research and debate. Low rectal tumors, which Claude F. Dixon and others have classically described as ‘the most controversial segment of the large intestine,” is a region of constant research and rapidly evolving procedures [10, 11]. In recent years, several new techniques aiming to preserve gastrointestinal continuity and improve both oncological and functional outcomes have emerged [12]. Techniques like the Transanal total mesorectal excision (TaTME) have been advocated by the 2017 European Society of Coloproctology (ESCP) collaborating group in their recent study [11]. LARS, which occurs in about 80% of the patients with low rectal tumors [13], is used to encompass a wide array of symptoms after sphincter preserving rectal surgery, including difficulty emptying the bowel, fecal urgency, and fecal incontinence [14]. Studies have also suggested that these LARS symptoms can last up to 15 years after surgery, thereby indicating that the decision to proceed with surgeries for low rectal cancers should weigh in these adverse effects [15].

Rectal cancer resection may be accompanied by the creation of a permanent or a temporary stoma. A temporary stoma is primarily created to reduce contamination from a leak at the primary anastomosis [16]. A post-anastomotic leak is one of the most dreaded complications due to an increased risk of mortality and morbidity for the patients [6]. Temporary stomas are usually reversed in 8 weeks (or sooner), generally following confirmation of satisfactory anastomotic healing by contrast studies [3]. In contrast, permanent stomas are most often created in situations where cancer involves the sphincter, when a negative margin cannot be achieved, in widely metastatic or unresectable diseases, and in prohibitive comorbidities of the patient that preclude anastomoses [17]. The most commonly associated complaint with these stomas is an inferior quality of life post-surgery [18, 19]. Reasons for this potentially include a patient's worsened body image, stoma-specific long and short-term complications, and limitations to daily activities, to name a few [3].

Our study found the presence of comorbidities and the elderly age group to have an association with having a permanent stoma after rectal cancer surgery. Ideally, the surgical approach for these patients should be made on a case-by-case basis, considering the extent of the disease, overall health condition of the patient, preoperative anorectal function, and the surgeon's experience dealing with such cases. Suboptimal disease control would result in local recurrence; local recurrence being the most consistent risk factor for permanent stoma in the literature [20]. Sometimes an anastomosis is technically doable, but even if that were the case, in some patients, we would not want to do an anastomosis in the off chance that they have a leak, which would be a life-ending event for them [20]. Also, elderly patients with a poor sphincter would have a more inferior quality of life if intestinal continuity restoration resulted in fecal incontinence.

Our analysis of socioeconomic factors revealed that individuals on Medicaid insurance or self-pay/uninsured had higher odds of receiving a permanent stoma than commercial insurance. Multiple studies have previously reported similar findings [21, 22]. Previous work has identified that Medicaid and self-pay/uninsured patients are less likely to receive cancer screening and tend to seek or receive care in the more advanced stages of the disease [5], making them more likely candidates for a permanent stoma. Similar aspects may contribute to the increased odds of receiving a permanent stoma in the Southwest of Florida compared to the Northeast, as previous disparities have been previously reported [23, 24]. Though there may be other possible factors involved, these are likely the main contributors for this cohort of patients.

The second part of our analysis focused on evaluating the factors that impact LOS for patients undergoing surgery for rectal cancer. We found these to be both patient and treatment-specific. In recent years, LOS has progressively decreased. While this aspect is promising and not wholly unexpected, it is multifactorial and is not easy to fully understand based solely on the data provided in this analysis. The literature has already widely described that patients with more comorbidities, metastatic cancer, or receiving open surgery often require a more extended stay in the hospital [25, 26]. These aspects directly impact patients' clinical recovery as longer perioperative treatments, multidisciplinary procedures or exams, and additional care are needed. Our analysis further reflected these facts.

Significant disparities were reported for African American patients, those covered by Medicaid as well as in various regions of the state. Race and insurance status are among the most common variables associated with healthcare disparities [27, 28]. These findings have been previously reported in different settings and conditions [29, 30]. Sharp et al. demonstrated that African American patients undergoing the creation of an intestinal stoma had a higher complication rate and a longer LOS than Caucasian patients [31]. Further, Hecht et al. suggested that race and socioeconomic status, such as low income, could predict who may suffer from poorer surgical outcomes [32]. Previous research has also demonstrated that there are differences associated with MIS use throughout the state and that access to care is not consistent in certain regions for patients of differing races or insurance status [23, 24]. However, it is not entirely clear how such factors interact and, ultimately, how disparities occur. Merely looking at the healthcare policies might not be enough since a more complex interaction of social, cultural, and psychological factors is also responsible. This interaction may differ from state to state, given the population's heterogeneity in many aspects, including racial distribution, median income, education level, and healthcare facilities distribution.

Limitations of this study include those involved in retrospective analyses of the database available on AHCA. For example, limited control of confounders, selection bias, and a high reliance on accurate data-keeping have to be considered. It is well known that types and cancer stages significantly influence the type of surgery performed on patients. Given the database’s nature, we could not adjust for disease stage except for patients with metastatic cancer. This inability to adjust for the cancer stage could represent a confounder as more advanced stages may require a more extended procedure and a justified need for a permanent stoma. Additionally, the lack of this information also limits our scope to accurately capture data about those temporary stomas, which were not reversed eventually. Since this critical information was unavailable to us, it forms a significant limitation of our study. The available dataset also did not provide information regarding postoperative complications and readmissions. The availability of this data would have helped form better associations with the LOS in these patients. Medicare patients younger than 65 were excluded from this study as these -few- patients are typically younger individuals with life-long debilities and constitute an entirely different population of patients. This study’s findings can be used to formulate prospective studies in the future to establish these associations further. Additionally, since this database represents patients only in Florida, it may have limited generalizability at the national level.

## Conclusions

Our analysis found a significant association between permanent stoma as an outcome of surgery for rectal cancer and socioeconomic factors such as having Medicaid insurance, being self-pay/uninsured, and residence in Southwest Florida. Clinical factors like metastatic disease, undergoing open surgery, and a higher number of comorbidities, and advanced age were also associated with having a permanent stoma after surgery. Furthermore, having a permanent stoma after surgery, male gender, African-American race, higher comorbidities, metastatic cancer, Medicaid insurance, Central, Southeast, Southwest, and West Central Florida were factors associated with a longer length of stay in the hospital after surgery. These findings emphasize the importance of improving our healthcare structure to reach those still deprived of it, as highlighted by our study. The gap in healthcare delivery shows no signs of narrowing, and as this study and others have shown, it is widening, a worrisome trend. Further studies are warranted to understand why these disparities provide all patients with the most optimal treatment available.

## Supplementary Information

**Additional file 1:** **Table S1** ICD10 Diagnosis and Procedure Codes.

## Data Availability

The datasets supporting the conclusions of this article are available in the Florida Agency for Health care Administration (AHCA) repository, which is available at https://www.floridahealthfinder.gov/Researchers/OrderData/order-data.aspx (8). A link to the dataset has been archived at https://perma.cc/58NZ-BBPR.

## Abbreviations

AHCA: Agency for health care administration
CI: Confidence interval
ERAS: Enhanced recovery after surgery
ICD: International classification of diseases
LOS: Length of Stay
MIS: Minimally invasive surgery
OR: Odds ratio
