# Supplementary material for: Permanent stoma: a quality outcome in treatment of rectal cancer and its impact on length of stay
Source: BMC Surg. 2021 Mar 25;21:163. doi: 10.1186/s12893-021-01166-7 (PMC7993534; doi:10.1186/s12893-021-01166-7)
Supplement: Supplementary file 1 — Additional file 1: Table S1 ICD10 Diagnosis and Procedure Codes. [file 12893_2021_1166_MOESM1_ESM.docx]

**Supplemental Table 1 – ICD10 Diagnosis and Procedure Codes**

**ICD 10 codes for colostomy**

| [0D1K0Z4](https://www.icd10data.com/ICD10PCS/Codes/0/D/1/K/0D1K0Z4) | Bypass Ascending Colon to Cutaneous | Open Approach |
| --- | --- | --- |
| [0D1K4Z4](https://www.icd10data.com/ICD10PCS/Codes/0/D/1/K/0D1K4Z4) | Bypass Ascending Colon to Cutaneous | Percutaneous Endoscopic Approach |
| [0D1K8Z4](https://www.icd10data.com/ICD10PCS/Codes/0/D/1/K/0D1K8Z4) | Bypass Ascending Colon to Cutaneous | Via Natural or Artificial Opening Endoscopic |
| [0D1K074](https://www.icd10data.com/ICD10PCS/Codes/0/D/1/K/0D1K074) | Bypass Ascending Colon to cutaneous | Open Approach |
| [0D1K474](https://www.icd10data.com/ICD10PCS/Codes/0/D/1/K/0D1K474) | Bypass Ascending Colon to cutaneous | Percutaneous Endoscopic Approach |
| [0D1K874](https://www.icd10data.com/ICD10PCS/Codes/0/D/1/K/0D1K874) | Bypass Ascending Colon to cutaneous | Via Natural or Artificial Opening Endoscopic |
| [0D1K0K4](https://www.icd10data.com/ICD10PCS/Codes/0/D/1/K/0D1K0K4) | Bypass Ascending Colon to cutaneous | Open Approach |
| [0D1K4K4](https://www.icd10data.com/ICD10PCS/Codes/0/D/1/K/0D1K4K4) | Bypass Ascending Colon to cutaneous | Percutaneous Endoscopic Approach |
| [0D1K8K4](https://www.icd10data.com/ICD10PCS/Codes/0/D/1/K/0D1K8K4) | Bypass Ascending Colon to cutaneous | Via Natural or Artificial Opening Endoscopic |
| [0D1K0J4](https://www.icd10data.com/ICD10PCS/Codes/0/D/1/K/0D1K0J4) | Bypass Ascending Colon to cutaneous | Open Approach |
| [0D1K3J4](https://www.icd10data.com/ICD10PCS/Codes/0/D/1/K/0D1K3J4) | Bypass Ascending Colon to cutaneous | Percutaneous Approach |
| [0D1K4J4](https://www.icd10data.com/ICD10PCS/Codes/0/D/1/K/0D1K4J4) | Bypass Ascending Colon to cutaneous | Percutaneous Endoscopic Approach |
| [0D1K8J4](https://www.icd10data.com/ICD10PCS/Codes/0/D/1/K/0D1K8J4) | Bypass Ascending Colon to cutaneous | Via Natural or Artificial Opening Endoscopic |
| [0D1H0Z4](https://www.icd10data.com/ICD10PCS/Codes/0/D/1/H/0D1H0Z4) | Bypass Cecum to Cutaneous | Open Approach |
| [0D1H4Z4](https://www.icd10data.com/ICD10PCS/Codes/0/D/1/H/0D1H4Z4) | Bypass Cecum to Cutaneous | Percutaneous Endoscopic Approach |
| [0D1H8Z4](https://www.icd10data.com/ICD10PCS/Codes/0/D/1/H/0D1H8Z4) | Bypass Cecum to Cutaneous | Via Natural or Artificial Opening Endoscopic |
| [0D1H074](https://www.icd10data.com/ICD10PCS/Codes/0/D/1/H/0D1H074) | Bypass Cecum to cutaneous | Open Approach |
| [0D1H474](https://www.icd10data.com/ICD10PCS/Codes/0/D/1/H/0D1H474) | Bypass Cecum to cutaneous | Percutaneous Endoscopic Approach |
| [0D1H874](https://www.icd10data.com/ICD10PCS/Codes/0/D/1/H/0D1H874) | Bypass Cecum to cutaneous | Via Natural or Artificial Opening Endoscopic |
| [0D1H0K4](https://www.icd10data.com/ICD10PCS/Codes/0/D/1/H/0D1H0K4) | Bypass Cecum to cutaneous | Open Approach |
| [0D1H4K4](https://www.icd10data.com/ICD10PCS/Codes/0/D/1/H/0D1H4K4) | Bypass Cecum to cutaneous | Percutaneous Endoscopic Approach |
| [0D1H8K4](https://www.icd10data.com/ICD10PCS/Codes/0/D/1/H/0D1H8K4) | Bypass Cecum to cutaneous | Via Natural or Artificial Opening Endoscopic |
| [0D1H0J4](https://www.icd10data.com/ICD10PCS/Codes/0/D/1/H/0D1H0J4) | Bypass Cecum to cutaneous | Open Approach |
| [0D1H3J4](https://www.icd10data.com/ICD10PCS/Codes/0/D/1/H/0D1H3J4) | Bypass Cecum to cutaneous | Percutaneous Approach |
| [0D1H4J4](https://www.icd10data.com/ICD10PCS/Codes/0/D/1/H/0D1H4J4) | Bypass Cecum to cutaneous | Percutaneous Endoscopic Approach |
| [0D1H8J4](https://www.icd10data.com/ICD10PCS/Codes/0/D/1/H/0D1H8J4) | Bypass Cecum to cutaneous | Via Natural or Artificial Opening Endoscopic |
| [0D1M0Z4](https://www.icd10data.com/ICD10PCS/Codes/0/D/1/M/0D1M0Z4) | Bypass Descending Colon to Cutaneous | Open Approach |
| [0D1M4Z4](https://www.icd10data.com/ICD10PCS/Codes/0/D/1/M/0D1M4Z4) | Bypass Descending Colon to Cutaneous | Percutaneous Endoscopic Approach |
| [0D1M8Z4](https://www.icd10data.com/ICD10PCS/Codes/0/D/1/M/0D1M8Z4) | Bypass Descending Colon to Cutaneous | Via Natural or Artificial Opening Endoscopic |
| [0D1M074](https://www.icd10data.com/ICD10PCS/Codes/0/D/1/M/0D1M074) | Bypass Descending Colon to cutaneous | Open Approach |
| [0D1M474](https://www.icd10data.com/ICD10PCS/Codes/0/D/1/M/0D1M474) | Bypass Descending Colon to cutaneous | Percutaneous Endoscopic Approach |
| [0D1M874](https://www.icd10data.com/ICD10PCS/Codes/0/D/1/M/0D1M874) | Bypass Descending Colon to cutaneous | Via Natural or Artificial Opening Endoscopic |
| [0D1M0K4](https://www.icd10data.com/ICD10PCS/Codes/0/D/1/M/0D1M0K4) | Bypass Descending Colon to cutaneous | Open Approach |
| [0D1M4K4](https://www.icd10data.com/ICD10PCS/Codes/0/D/1/M/0D1M4K4) | Bypass Descending Colon to cutaneous | Percutaneous Endoscopic Approach |
| [0D1M8K4](https://www.icd10data.com/ICD10PCS/Codes/0/D/1/M/0D1M8K4) | Bypass Descending Colon to cutaneous | Via Natural or Artificial Opening Endoscopic |
| [0D1M0J4](https://www.icd10data.com/ICD10PCS/Codes/0/D/1/M/0D1M0J4) | Bypass Descending Colon to cutaneous | Open Approach |
| [0D1M3J4](https://www.icd10data.com/ICD10PCS/Codes/0/D/1/M/0D1M3J4) | Bypass Descending Colon to cutaneous | Percutaneous Approach |
| [0D1M4J4](https://www.icd10data.com/ICD10PCS/Codes/0/D/1/M/0D1M4J4) | Bypass Descending Colon to cutaneous | Percutaneous Endoscopic Approach |
| [0D1M8J4](https://www.icd10data.com/ICD10PCS/Codes/0/D/1/M/0D1M8J4) | Bypass Descending Colon to cutaneous | Via Natural or Artificial Opening Endoscopic |
| [0D1N0Z4](https://www.icd10data.com/ICD10PCS/Codes/0/D/1/N/0D1N0Z4) | Bypass Sigmoid Colon to Cutaneous | Open Approach |
| [0D1N4Z4](https://www.icd10data.com/ICD10PCS/Codes/0/D/1/N/0D1N4Z4) | Bypass Sigmoid Colon to Cutaneous | Percutaneous Endoscopic Approach |
| [0D1N8Z4](https://www.icd10data.com/ICD10PCS/Codes/0/D/1/N/0D1N8Z4) | Bypass Sigmoid Colon to Cutaneous | Via Natural or Artificial Opening Endoscopic |
| [0D1N074](https://www.icd10data.com/ICD10PCS/Codes/0/D/1/N/0D1N074) | Bypass Sigmoid Colon to cutaneous | Open Approach |
| [0D1N474](https://www.icd10data.com/ICD10PCS/Codes/0/D/1/N/0D1N474) | Bypass Sigmoid Colon to cutaneous | Percutaneous Endoscopic Approach |
| [0D1N874](https://www.icd10data.com/ICD10PCS/Codes/0/D/1/N/0D1N874) | Bypass Sigmoid Colon to cutaneous | Via Natural or Artificial Opening Endoscopic |
| [0D1N0K4](https://www.icd10data.com/ICD10PCS/Codes/0/D/1/N/0D1N0K4) | Bypass Sigmoid Colon to cutaneous | Open Approach |
| [0D1N4K4](https://www.icd10data.com/ICD10PCS/Codes/0/D/1/N/0D1N4K4) | Bypass Sigmoid Colon to cutaneous | Percutaneous Endoscopic Approach |
| [0D1N8K4](https://www.icd10data.com/ICD10PCS/Codes/0/D/1/N/0D1N8K4) | Bypass Sigmoid Colon to cutaneous | Via Natural or Artificial Opening Endoscopic |
| [0D1N0J4](https://www.icd10data.com/ICD10PCS/Codes/0/D/1/N/0D1N0J4) | Bypass Sigmoid Colon to cutaneous | Open Approach |
| [0D1N3J4](https://www.icd10data.com/ICD10PCS/Codes/0/D/1/N/0D1N3J4) | Bypass Sigmoid Colon to cutaneous | Percutaneous Approach |
| [0D1N4J4](https://www.icd10data.com/ICD10PCS/Codes/0/D/1/N/0D1N4J4) | Bypass Sigmoid Colon to cutaneous | Percutaneous Endoscopic Approach |
| [0D1N8J4](https://www.icd10data.com/ICD10PCS/Codes/0/D/1/N/0D1N8J4) | Bypass Sigmoid Colon to cutaneous | Via Natural or Artificial Opening Endoscopic |
| [0D1L0Z4](https://www.icd10data.com/ICD10PCS/Codes/0/D/1/L/0D1L0Z4) | Bypass Transverse Colon to Cutaneous | Open Approach |
| [0D1L4Z4](https://www.icd10data.com/ICD10PCS/Codes/0/D/1/L/0D1L4Z4) | Bypass Transverse Colon to Cutaneous | Percutaneous Endoscopic Approach |
| [0D1L8Z4](https://www.icd10data.com/ICD10PCS/Codes/0/D/1/L/0D1L8Z4) | Bypass Transverse Colon to Cutaneous | Via Natural or Artificial Opening Endoscopic |
| [0D1L074](https://www.icd10data.com/ICD10PCS/Codes/0/D/1/L/0D1L074) | Bypass Transverse Colon to cutaneous | Open Approach |
| [0D1L474](https://www.icd10data.com/ICD10PCS/Codes/0/D/1/L/0D1L474) | Bypass Transverse Colon to cutaneous | Percutaneous Endoscopic Approach |
| [0D1L874](https://www.icd10data.com/ICD10PCS/Codes/0/D/1/L/0D1L874) | Bypass Transverse Colon to cutaneous | Via Natural or Artificial Opening Endoscopic |
| [0D1L0K4](https://www.icd10data.com/ICD10PCS/Codes/0/D/1/L/0D1L0K4) | Bypass Transverse Colon to cutaneous | Open Approach |
| [0D1L4K4](https://www.icd10data.com/ICD10PCS/Codes/0/D/1/L/0D1L4K4) | Bypass Transverse Colon to cutaneous | Percutaneous Endoscopic Approach |
| [0D1L8K4](https://www.icd10data.com/ICD10PCS/Codes/0/D/1/L/0D1L8K4) | Bypass Transverse Colon to cutaneous | Via Natural or Artificial Opening Endoscopic |
| [0D1L0J4](https://www.icd10data.com/ICD10PCS/Codes/0/D/1/L/0D1L0J4) | Bypass Transverse Colon to cutaneous | Open Approach |
| [0D1L3J4](https://www.icd10data.com/ICD10PCS/Codes/0/D/1/L/0D1L3J4) | Bypass Transverse Colon to cutaneous | Percutaneous Approach |
| [0D1L4J4](https://www.icd10data.com/ICD10PCS/Codes/0/D/1/L/0D1L4J4) | Bypass Transverse Colon to cutaneous | Percutaneous Endoscopic Approach |

**ICD 10 codes for ileostomy**

| [0D1B0Z4](https://www.icd10data.com/ICD10PCS/Codes/0/D/1/B/0D1B0Z4) | Bypass Ileum to Cutaneous | Open Approach |
| --- | --- | --- |
| [0D1B4Z4](https://www.icd10data.com/ICD10PCS/Codes/0/D/1/B/0D1B4Z4) | Bypass Ileum to Cutaneous | Percutaneous Endoscopic Approach |
| [0D1B8Z4](https://www.icd10data.com/ICD10PCS/Codes/0/D/1/B/0D1B8Z4) | Bypass Ileum to Cutaneous | Via Natural or Artificial Opening Endoscopic |
| [0D1B074](https://www.icd10data.com/ICD10PCS/Codes/0/D/1/B/0D1B074) | Bypass Ileum to cutaneous | Open Approach |
| [0D1B474](https://www.icd10data.com/ICD10PCS/Codes/0/D/1/B/0D1B474) | Bypass Ileum to cutaneous | Percutaneous Endoscopic Approach |
| [0D1B874](https://www.icd10data.com/ICD10PCS/Codes/0/D/1/B/0D1B874) | Bypass Ileum to cutaneous | Via Natural or Artificial Opening Endoscopic |
| [0D1B0K4](https://www.icd10data.com/ICD10PCS/Codes/0/D/1/B/0D1B0K4) | Bypass Ileum to cutaneous | Open Approach |
| [0D1B4K4](https://www.icd10data.com/ICD10PCS/Codes/0/D/1/B/0D1B4K4) | Bypass Ileum to cutaneous | Percutaneous Endoscopic Approach |
| [0D1B8K4](https://www.icd10data.com/ICD10PCS/Codes/0/D/1/B/0D1B8K4) | Bypass Ileum to cutaneous | Via Natural or Artificial Opening Endoscopic |
| [0D1B0J4](https://www.icd10data.com/ICD10PCS/Codes/0/D/1/B/0D1B0J4) | Bypass Ileum to cutaneous | Open Approach |
| [0D1B3J4](https://www.icd10data.com/ICD10PCS/Codes/0/D/1/B/0D1B3J4) | Bypass Ileum to cutaneous | Percutaneous Approach |
| [0D1B4J4](https://www.icd10data.com/ICD10PCS/Codes/0/D/1/B/0D1B4J4) | Bypass Ileum to cutaneous | Percutaneous Endoscopic Approach |
| [0D1B8J4](https://www.icd10data.com/ICD10PCS/Codes/0/D/1/B/0D1B8J4) | Bypass Ileum to cutaneous | Via Natural or Artificial Opening Endoscopic |

**ICD 10 codes for surgical resection of the rectum**

| [0DBP0ZZ](https://www.icd10data.com/ICD10PCS/Codes/0/D/B/P/0DBP0ZZ) | Excision of Rectum | Open Approach |
| --- | --- | --- |
| [0DBP3ZZ](https://www.icd10data.com/ICD10PCS/Codes/0/D/B/P/0DBP3ZZ) | Excision of Rectum | Percutaneous Approach |
| [0DBP4ZZ](https://www.icd10data.com/ICD10PCS/Codes/0/D/B/P/0DBP4ZZ) | Excision of Rectum | Percutaneous Endoscopic Approach |
| [0DBP7ZZ](https://www.icd10data.com/ICD10PCS/Codes/0/D/B/P/0DBP7ZZ) | Excision of Rectum | Via Natural or Artificial Opening |
| [0DBP8ZZ](https://www.icd10data.com/ICD10PCS/Codes/0/D/B/P/0DBP8ZZ) | Excision of Rectum | Via Natural or Artificial Opening Endoscopic |

| [0DTP0ZZ](https://www.icd10data.com/ICD10PCS/Codes/0/D/T/P/0DTP0ZZ) | Resection of Rectum | Open Approach |
| --- | --- | --- |
| [0DTP4ZZ](https://www.icd10data.com/ICD10PCS/Codes/0/D/T/P/0DTP4ZZ) | Resection of Rectum | Percutaneous Endoscopic Approach |
| [0DTP7ZZ](https://www.icd10data.com/ICD10PCS/Codes/0/D/T/P/0DTP7ZZ) | Resection of Rectum | Via Natural or Artificial Opening |
| [0DTP8ZZ](https://www.icd10data.com/ICD10PCS/Codes/0/D/T/P/0DTP8ZZ) | Resection of Rectum | Via Natural or Artificial Opening Endoscopic |

**ICD 10 codes for rectal cancer diagnosis**

[C20](https://www.icd10data.com/ICD10CM/Codes/C00-D49/C15-C26/C20-/C20) Malignant neoplasm of rectum

[C19](https://www.icd10data.com/ICD10CM/Codes/C00-D49/C15-C26/C19-/C19) Malignant neoplasm of rectosigmoid junction
